# The Developmental Trajectory and Influencing Factors of Self-Concept Clarity in Chinese Adolescents: A Latent Transition Analysis

**DOI:** 10.3390/bs15091257

**Published:** 2025-09-14

**Authors:** Yang Yang, Ying Zou, Yin Qiu, Jianyong Yang

**Affiliations:** 1 Mental Health Education Center, Huanghuai University, Zhumadian 463000, China; 2 Mental Health Education Center, Wuhan College, Wuhan 430212, China; 3 Key Laboratory of Adolescent Cyberpsychology and Behavior (CCNU), Ministry of Education, Wuhan 430079, China; 4 Key Laboratory of Human Development and Mental Health of Hubei Province, School of Psychology, Central China Normal University, Wuhan 430079, China

**Keywords:** self-concept clarity, parent–child relationships, peer relationships, latent transition analysis (LTA)

## Abstract

This study used latent transition analysis to explore the categories of self-concept clarity transformation and their influencing factors among adolescents. A total of 3010 adolescents (*Mage* = 17.56, *SD* = 2.61, ranging from 12 to 23 years, 41.23% males) were longitudinally tracked, with assessments of their self-concept clarity conducted three times over half a year. Results showed that (1) there existed heterogeneity in the adolescents’ self-concept clarity, with three distinct profiles identified at each time point; and (2) adolescents’ self-concept clarity exhibited different transition trajectories. The group with high self-concept clarity revealed higher stability, with those in the low self-concept clarity group tending to shift towards either the moderate self-concept clarity group or the high self-concept clarity group. (3) Parent–child relationships had a more stable impact on adolescents’ self-concept clarity subgroups, while peer relationships played a less consistent role. The study advances our understanding of the potential changes in the development of adolescent self-concept clarity profiles in conjunction with the impact of peer relationships and parent–child relationships, but also offers a basis for classification description and intervention practices in enhancing adolescents’ self-concept clarity.

## 1. Introduction

Self-concept clarity reflects the degree of certainty and clarity in the content of one’s self-concept, along with its internal consistency and stability over time (Campbell, 1990; Campbell et al., 1996, p. 141). In other words, it refers to the extent to which various aspects of the self are coherently integrated into a well-defined and unified whole (Jiang et al., 2020). Specifically, self-concept clarity is defined as (1) the extent to which individuals have a clearly defined understanding of their traits and beliefs, (2) the internal consistency and congruence among various aspects of the self, and (3) the perceived stability and coherence of these traits across different situations and over time. Self-concept clarity, as a cognitive aspect of self-concept integrity (Campbell et al., 1996; Lodi-Smith et al., 2017), exerts a powerful influence on an individual’s thoughts, feelings, and actions (Levey et al., 2019; Posavac & Posavac, 2020).

The formation of an integrated self-concept and identity constitutes a critical developmental task during adolescence (Gilmore & Meersand, 2014; Suszek et al., 2018). For adolescents, a clear self-concept is an important indicator of personal personality maturation (Lodi-Smith & DeMarree, 2017) and can provide a solid foundation for psychological and social adaptation (Coutelle et al., 2020; Landa, 2021). Self-concept clarity is vital for adolescents’ mental health, subjective well-being, and psychological adjustment (Lodi-Smith & DeMarree, 2017). However, adolescents are in a period of extensive changes in physiology, cognition, emotions, and social relationships, so the development of their self-concept clarity may exhibit more complex profiles. Identifying the differentiation profiles of adolescents’ self-concept clarity, along with their developmental transformation profiles, holds significant theoretical and practical value. It not only provides a deeper understanding of the developmental trajectories of adolescents’ self-concept but also informs the design of targeted interventions for those exhibiting lower levels of self-concept clarity. Developmental transition patterns and factors influencing self-concept clarity were examined through the person-centered study.

### 1.1. The Stability and Dynamism of Self-Concept Clarity

Self-concept clarity tends to be stable and is viewed as a trait-like construct. According to a longitudinal meta-analysis including 70 effect sizes, self-concept clarity demonstrated a rank-order stability coefficient of 0.75 (Lodi-Smith & Crocetti, 2017), indicating a level of stability comparable to that of personality traits across the lifespan (Roberts & DelVecchio, 2000). The rank-order stability coefficient was found to decline with longer intervals between measurements (r = −0.58), while showing a positive association with age (r = 0.20) (Lucas & Donnellan, 2011; Roberts & DelVecchio, 2000). Longitudinal studies have demonstrated that the average level of self-concept clarity tends to remain relatively stable over varying time intervals (Xiang et al., 2023; Zhou et al., 2023). For example, Xiang et al. (2023) explored whether self-concept clarity increased over time among Chinese adolescents (*N* = 2001) and found no significant mean-level change across the three waves. However, other longitudinal studies have also highlighted that self-concept clarity exhibits an upward developmental trajectory. Crocetti et al. (2016) conducted a six-wave longitudinal study spanning six years with two independent samples of Dutch adolescents (*N*_1_ = 244, *N*_2_ = 497). Findings from both cohorts consistently revealed a nonlinear developmental trajectory of self-concept clarity, characterized by gradual increases over time. Similarly, Weber et al. (2023) carried out monthly assessments over an eight-month period with two samples of young adults (*N*_1_ = 460; *N*_2_ = 412) and reported small to moderate increases in self-concept clarity.

Previous research has primarily focused on the trend of changes in self-concept clarity, reflecting average developmental trajectories at the group level while neglecting individual differences in the development of self-concept clarity. Person–context interaction theory suggests that the fundamental characteristic of human development that might be caused by complex environments is diversity. Individuals with differences tend to cluster together due to similarities in certain psychological and behavioral traits, presenting themselves as more stable “types” (Magnusson & Stattin, 1998). Nevertheless, few studies have investigated the differentiation of self-concept clarity patterns. Three different self-concept clarity subgroups were found by Xiang et al. (2021) after surveying 2792 teenagers: one with a low level of self-concept clarity, one with a moderate level, and one with a high level. Similarly, Yang et al. (2025) surveyed 2288 adolescents and also found consistent results. However, these studies were cross-sectional and did not further explore how the differentiation patterns of adolescents’ self-concept clarity transform across different time points, nor did they conduct an in-depth analysis of the potential factors that may influence these transitional trajectories. By employing a longitudinal design and utilizing latent transition analysis (LTA), the study systematically tracks the developmental trajectories of different self-concept clarity profiles in adolescents and explores potential influencing factors.

### 1.2. The Impact of Peer Relationships and Parent–Child Relationships on Self-Concept Clarity

The ecological theory underscores that individual development is gradually constructed within a multi-level social environment (Bronfenbrenner, 2000), which offers a useful framework for comprehending how self-concept clarity is formed. Self-concept clarity refers to the individual’s clear, consistent, and stable understanding of themselves, which is directly influenced by a multilayered social environment. Research shows that environmental factors play an important role in shaping the clarity of self-concept (Du et al., 2023). For example, living abroad would enhance individuals’ self-concept clarity (Adam et al., 2018). Additionally, social construction theory also posits that the self is gradually constructed and formed through social interpersonal interactions and social exchanges. In other words, the emergence of the self relies on relationships with others within the discursive environment. Thus, to some extent, the self is relational. From this perspective, the self is shaped through social interactions and an individual’s relationships with others (Gergen, 1985).

According to the person–context interaction theory (Magnusson & Stattin, 1998) and ecological theory (Bronfenbrenner, 2000), peer relationships and parent–child relationships might have an impact on differentiation profiles and shifts in the clarity of their self-concept. Both peer relationships and parent–child relationships are key forms of social interaction within the adolescent microsystem, playing a crucial role in the development of self-concept. Peer relationships provide an important social context for adolescents to construct their self-concept. Through interactive experiences and by referencing peers’ evaluations, adolescents assess their academic abilities and social skills, thereby forming related self-concepts. For example, X. Zhang et al. (2015) discovered that the quality of friendships among high school students influenced their self-concept clarity, with higher quality friendships associated with higher self-concept clarity. The relationships between parents and children are foundational in shaping adolescents’ self-concept. The parent–child relationships have a buffering or protective effect on adolescents under stress. For example, parental control has been found to affect adolescents’ depressive symptoms, potentially through its impact on self-concept clarity (Li et al., 2022). Additionally, family functioning and open communication with parents demonstrated to have a positive effect on teenagers’ clarity of self-concept over time (Van Dijk et al., 2014; Zhou et al., 2023). Meanwhile, self-concept clarity demonstrated generational transmission, whereby parents’ self-concept clarity affected adolescents’ self-concept clarity (Crocetti et al., 2016). Furthermore, evidence suggests that peer relationships and parent–child relationships simultaneously buffer the negative effects of online literature reading on self-concept clarity (D. Zhang, 2018).

In summary, previous research has predominantly explored the influencing factors of self-concept clarity from a variable-centered perspective, focusing on the independent or interactive effects of different variables on self-concept clarity. However, this perspective often overlooks individual differences and the diverse developmental pathways individuals exhibit in various social contexts. In recent years, as statistical methods advanced, researchers began incorporating individual-centered analysis techniques such as latent potential analysis (LPA) or latent transition analysis (LTA) to identify distinct profiles of self-concept clarity development in individual groups. This approach facilitates the revelation of the complex evolutionary processes of self-concepts under different social interaction contexts, enabling a more comprehensive understanding of how individuals construct self-concept clarity within societal systems.

### 1.3. The Current Study

The study investigates how adolescents’ self-concept clarity differentiates and changes over six months. It tracks a large sample of adolescents through three waves of surveys. Latent profile analysis and latent transition analysis are used to analyze the data. It further explores the predictive roles of peer relationships and parent–child relationships on different adaptive subgroups and their transitions. Based on the theoretical frameworks and prior research outlined above, this study proposes the following hypotheses:

**H_1_.**  *Adolescents can be categorized into distinct profiles defined by varying levels of self-concept clarity at different times.*

**H_2_.**  *Adolescents’ self-concept clarity would display different transition patterns among profiles across time.*

**H_3_.**  *Peer relationships and parent–child relationships would significantly influence adolescents’ transitions between different latent profiles of self-concept clarity over time.*

## 2. Materials and Methods

### 2.1. Participants and Procedure

Cluster sampling was used to gather study data in compliance with the ethical principle of informed consent. After obtaining approval from the schools, students voluntarily took part in the survey. The study adopted a six-month longitudinal design, with three waves of data collection (T_1_, T_2_, and T_3_) conducted at three-month intervals. A total of 3010 adolescents were included, among whom 2426 completed all three waves of the survey, and 584 completed two waves. Of these, 1219 were secondary school students (*M*_age_ = 14.79, *SD* = 1.57), including 549 males (45.04%) and 670 females (54.96%). A total of 1791 were college students (*M*_age_ = 19.45, *SD* = 1.00), including 692 males (38.64%) and 1099 females (61.36%). The first survey (T_1_) collected 3010 valid questionnaires; three months later, the second survey (T_2_) collected 2750 valid questionnaires; and six months later, the third survey (T_3_) collected 2683 valid questionnaires, with attrition rates of 8.63% and 10.86%, respectively.

### 2.2. Measures

The questionnaires were all rated on a 5-point Likert scale (1 = strongly disagree, 5 = strongly agree).

Self-Concept Clarity: The study used the revised version of the self-concept clarity scale by Niu et al. (2016), developed by Campbell et al. (1996). The scale includes 12 items (sample item: “If I were to describe my personality, perhaps my description would be different every day”). Higher scores indicated higher self-concept clarity. It demonstrated good internal consistency, with Cronbach’s α of 0.82, 0.85, and 0.86, respectively.

Peer Relationships: The study used two subscales of the revised version of the Friendship Quality Inventory by Fan and Fang (2004). Two subscales were employed, including companionship (sample item: “Doing enjoyable things together with your friend”) and intimacy (sample item: “Sharing things with your friend that you wouldn’t tell anyone else”). Higher scores indicated better peer relationships. The validity and reliability of the scale in Chinese adolescents have been validated (D. Zhang, 2018). The study demonstrated good internal consistency, with Cronbach’s α of 0.80, 0.81, and 0.79, respectively.

Parent–Child Relationships: The study used the revised version of the Parental Closeness Measure originally developed by Stepney et al. (2015). The scale has 10 items (sample item: “I feel that I can talk to my parents about almost anything”). Higher scores reflected a greater sense of closeness with parents. Previous studies have confirmed the scale’s reliability and validity (Yu & Chang, 2018). The study demonstrated good internal consistency, with Cronbach’s α of 0.93, 0.94, and 0.94, respectively.

### 2.3. Data Analysis

To identify potential patterns of self-concept clarity, the study conducted latent profile analysis based on items of the self-concept clarity scale, consistent with prior research (Xiang et al., 2021; Yang et al., 2025). Preliminary data analysis was conducted using SPSS 25.0, including descriptive statistical analysis and correlation analysis. Based on the results of the correlation analysis, latent profile analysis and latent transition analysis were performed using Mplus 8.0. Model evaluation involves several key indicators. First, entropy reflects the precision of classification, with values ranging from 0 to 1. Higher values denote greater accuracy; specifically, values above 0.8 indicate over 90% classification accuracy (Jung & Wickrama, 2008). Second, information-based criteria such as AIC, BIC, and adjusted BIC provide estimates of model fit, where lower values generally indicate superior fit. Third, likelihood ratio tests—notably, the Vuong–Lo–Mendell–Rubin (VLMR-LRT) and bootstrap likelihood ratio test (BLRT)—are used to compare models with K versus K − 1 classes, helping to determine whether the inclusion of an additional class leads to a significantly better-fitting model. Ultimately, selection of the optimal class solution should also consider theoretical frameworks and previous empirical evidence (Nylund et al., 2007).

## 3. Results

### 3.1. Descriptive Statistics and Correlation Analysis

The descriptive statistics of each variable are presented in Table 1. Peer relationships, parent–child relationships, and self-concept clarity were all significantly correlated with each other both within the same time and across different times.

### 3.2. Differentiation Profiles of Adolescent Self-Concept Clarity: Latent Profile Analysis

To identify potential patterns, this study conducted latent profile analysis based on self-concept clarity items, consistent with prior research (Xiang et al., 2021; Yang et al., 2025), to explore different profiles from an individual-centered perspective. The study established 2–6 latent profile models at three time points, with fit indices shown in Table 2. At Time 1 (T1), the results showed that the three-profile solution yielded the highest entropy value (Clark & Muthén, 2009), the most substantial reductions in AIC, BIC, and aBIC, suggesting a turning point in model fit and supporting the appropriateness of the three-profile model (Nylund-Gibson et al., 2023). With a range of 0.91 to 0.96 for the probability of each observation being matched to a latent profile, the classification accuracy was high. At Time 2 (T2), although the entropy of the three-profile model was lower than that of the two-profile model, the three-profile model showed the greatest reductions in AIC, BIC, and aBIC, indicating a turning point (Nylund-Gibson et al., 2023). Moreover, classification probabilities for each observation in the three-profile model ranged from 0.91 to 0.94, suggesting a high level of classification accuracy. At Time 3 (T_3_), although the changes in entropy of the three-profile model were not higher compared to other models, the probability range for each observation being categorized into each latent profile is 0.90 to 0.96, showing a high classification accuracy. The reductions in AIC, BIC, and aBIC were greatest at three profiles. Hence, across the three time points, the optimal latent profile model was the three-profile solution.

**Figure 1:**
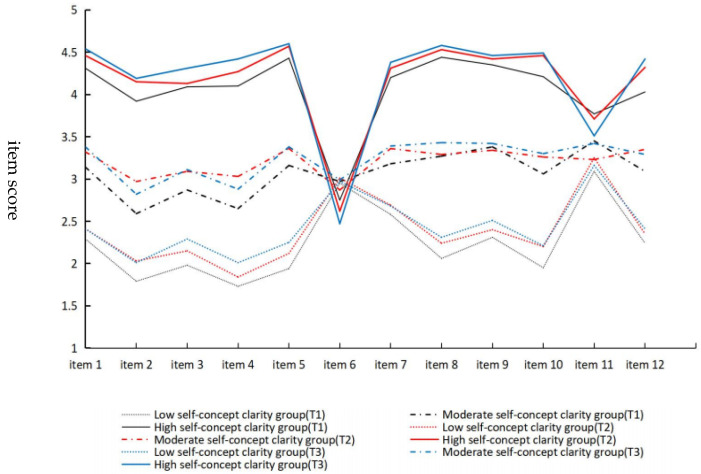
Latent profiles of adolescent self-concept clarity.

Latent potential analysis model results across three time points were conducted to describe and name different subgroups. As depicted in Figure 1, the three subgroups were relatively similar across the three time points. The first subgroup of adolescents was characterized by lower scores on the self-concept clarity scale, accounting for 24.55%, 21.20%, and 10.51% at T_1_, T_2_, and T_3_, respectively. This group was classified as the low self-concept clarity group. The second subgroup of adolescents was characterized by moderate scores on the self-concept clarity scale, accounting for 61.26%, 53.35% and 60.34% at T_1_, T_2_, and T_3_, respectively. This group was classified as the moderate self-concept clarity group. The third subgroup of adolescents was characterized by high scores on the self-concept clarity scale except item 6, accounting for 14.19%, 25.45%, and 29.15% at T_1_, T_2_, and T_3_, respectively. This group was classified as the high self-concept clarity group.

### 3.3. The Transformation of the Differentiation Profile of Adolescents’ Self-Concept Clarity

The number and characteristics of the subgroups were largely consistent across the three time points. Therefore, the study conducted latent transition analysis to examine changes within the three subgroups over time. Table 3 displays the probability of adolescents in different subgroups remaining within their initial groups or transitioning to other subgroups at the next time point. The bold diagonal line in the transition matrix represents the probability of adolescents maintaining their original potential profile.

The results showed the high self-concept clarity group had the highest profile stability from T_1_ to T_2_, with a transition probability of 79.40%. Although the probability decreased slightly from T_2_ to T_3_ (73.70%), it still reflected a relatively high level of stability. The moderate self-concept clarity group also maintained its original profile with relatively higher probabilities (66.50% and 78.30%), while the group also tended to transform into the high group, being 25.90% and 16.80%, respectively. The low self-concept clarity group tended to transform into the moderate group, being 42.40% and 59.40%, respectively. But the probability of transition from the low self-concept clarity group to the high self-concept clarity group was relatively small.

### 3.4. The Impact of Peer Relationships and Parent–Child Relationships on Subgroup Transitions in Adolescents’ Self-Concept Clarity

To further explore the role of peer relationships and parent–child relationships in the subgroup transitions of adolescents’ self-concept clarity, adolescents who remained in the original subgroup served as the reference group. A multiple logistic regression was performed to calculate the odds ratios. The odds ratio reflects the probability of adolescents transitioning to other subgroups compared to the probability of remaining in the original subgroup. If the odds ratio is more than 1, it means that under the influence of peer relationships and parent–child relationships, the likelihood of adolescents making that transition increases, while a ratio less than 1 indicates a decrease in probability. The results are shown in Table 4.

Taking the transformation from the low self-concept clarity group to the low self-concept clarity group as the reference, the influence of peer relationships and parent–child relationships on the transformation of self-concept clarity was not significant from T_1_ to T_2_, or from T_2_ to T_3_. Taking the transformation from the moderate self-concept clarity group to the moderate self-concept clarity group as the reference, increased parent–child relationships reduced the likelihood of adolescents transitioning from the moderate self-concept clarity group to the low self-concept clarity group (B = −0.45, *SE* = 0.14, *p* < 0.001, OR = 0.64) from T_1_ to T_2_. On the other hand, as parent–child relationships increased, adolescents were more likely to transition from the moderate self-concept clarity group to the high group (B = 0.48/0.50, *SE* = 0.08/0.12, *p* < 0.001, OR = 1.61/1.64) from T_1_/T_2_ to T_2_/T_3_. Similarly, as the peer relationships increased, the probability of adolescents transforming from the moderate self-concept clarity group to the high group increased (B = −0.25, *SE* = 0.12, *p* < 0.001, OR = 1.28) from T_2_ to T_3_. Finally, taking the transformation from the high self-concept clarity group to the high self-concept clarity group as the reference, increased parent–child relationships reduced the likelihood of transitions from the high self-concept clarity group to the moderate group (B = −0.64/−0.29, *SE* = 0.20/0.13, *p* < 0.001, OR = 0.53/0.75) T_1_/T_2_ to T_2_/T_3_.

## 4. Discussion

### 4.1. Profiles of Adolescent Self-Concept Clarity

Through latent profile analysis, the present study identified three distinct subgroups of adolescents based on self-concept clarity: the low self-concept clarity group, the moderate self-concept clarity group, and the high self-concept clarity group. Moreover, this classification demonstrated temporal stability across multiple time points. The results are in line with findings from prior studies (Xiang, 2022; Yang et al., 2025), which examined individual differences in cross-sectional studies. Expanding on these results, the current study extends the results to multiple time points and reveals similarities in individual differences over time. Among the three time points, the subgroup with the largest number of adolescents belonged to the moderate self-concept clarity group, followed by those with low self-concept clarity, and the group with the highest self-concept clarity had the smallest proportion. The classification of “type” further supports the person–context interaction theory (Magnusson & Stattin, 1998), suggesting that the development of self-concept clarity displays a range of distinct characteristics. Adolescents may go through various identification stages as they explore who they are. The group with a low level of self-concept clarity may be experiencing “identity confusion” or “identity moratorium,” whereas the group with a high level of self-concept clarity may be experiencing “identity achievement.” The moderate self-concept clarity group, on the other hand, might be in a transitional phase. At three time points, more than half of the adolescents were in the moderate self-concept clarity group, while nearly 10–20% of the adolescents were in the low self-concept clarity group. These findings suggest that, for adolescents, self-concept is still in a formative stage, and they continue to confront the developmental challenge of establishing their self-identity (Xiang, 2022).

### 4.2. Latent Transitions of Self-Concept Clarity

Overall, the results underscored a distinct pattern of profile stability across self-concept clarity levels, with adolescents in the low clarity group exhibiting the most instability, followed by those in the moderate group, and the highest stability observed among those in the high group. This pattern is partly supported by the fact that adolescence represents a crucial phase in the formation of self and identity, characterized by significant fluctuations in self-attitudes and perceptions (Erikson, 1968; Waterman, 1982). Adolescents are in the stage of “identity confusion”, particularly when navigating the challenges of seeking self-identification. This instability is exacerbated by the fluctuating nature of self-concept during adolescence, a critical period for the exploration and consolidation of one’s identity (Lodi-Smith & DeMarree, 2017).

The high self-concept clarity group showed low transition probabilities across three time points, with retention rates of 79.40% from T_1_ to T_2_ and 73.70% from T_2_ to T_3_. This suggests that adolescents in this group exhibit a high degree of stability in their self-concept, with little or no significant changes occurring over time. This might imply that adolescents with high self-concept clarity tend to have more solid self-awareness, a stronger sense of identity, and lower sensitivity to changes in their self-concept. Adolescents with a high level of self-concept clarity have been found to have higher subjective well-being (Xiang et al., 2021) and meaning in life (Yang et al., 2025) in prior studies. In contrast, the low self-concept clarity group showed high transition probabilities across three time points, with rates of 55.60% from T_1_ to T_2_ and 62.40% from T_2_ to T_3_. The transition from a low self-concept clarity group to a moderate or high self-concept clarity group might reflect an adaptive process, where individuals gain more coherent and stable understandings of themselves. This is consistent with longitudinal studies showing that self-concept clarity tends to increase gradually. Meanwhile, the results reveal that individuals in the high self-concept clarity group also transition to either the moderate or low self-concept clarity groups, while those in the moderate self-concept clarity group shift towards the low self-concept clarity group. These transitions might suggest a maladaptive process, pointing to significant fluctuations in self-concept clarity that reflect underlying instability in identity formation. Such shifts could indicate periods of psychological vulnerability, where external factors such as changes in social roles (Su et al., 2021), the disruption of close relationships (Slotter et al., 2010), or daily negative life (Nezlek & Plesko, 2001) could result in decreased self-concept clarity. This transformation highlights the fluctuations of self-concept clarity, suggesting that even individuals with initially high self-concept clarity are not immune to identity struggles, and underscores the need for targeted interventions to promote stability in self-identity during adolescence.

### 4.3. Predictive Effects of Peer Relationships and Parent–Child Relationships on Transitions Between Latent Profiles

The study revealed that parent–child relationships were a relatively robust factor influencing transitions in adolescent self-concept clarity. Peer relationships were found to predict transitions only from the moderate self-concept clarity group to the high self-concept clarity group from T_2_ to T_3_. The findings provide support for the ecological systems theory, which emphasizes the significant influence of microsystems (such as family and peers) on psychological development (Bronfenbrenner, 2000). It also aligns with the social constructionist perspective, which posits that individuals gradually construct their self-concept through interactions with others (Gergen, 1985). The findings are consistent with previous variable-centered research, which has shown that both peer relationships and parent–child relationships are associated with higher levels of self-concept clarity (X. Zhang et al., 2015; Zhou et al., 2023).

Findings suggest that compared to peer relationships, parent–child relationships have a more stable and widespread impact on adolescent self-concept clarity, particularly in maintaining or enhancing higher levels of self-concept clarity. It has once again been confirmed that parent–child relationships are the foundation for adolescents’ positive development (Parke & Ladd, 2016). Parent–child relationships are closely associated with long-term stability and emotional support, such as open communication with parents (Van Dijk et al., 2014), which helps adolescents form a more consistent and clear self-cognitive structure. Meanwhile, the clarity of parents’ self-concept played a significant role in shaping adolescents’ self-concept clarity (Crocetti et al., 2016). Moreover, studies have indicated that parent–child relationships serve as the foundation for the development of peer relationships (X. Zhang et al., 2019). The internal schemas formed through secure attachment with parents during early childhood influence the subsequent establishment of relationships with peers (Bowlby, 1982). Although peer relationships are particularly important during adolescence, they are more influenced by external factors. For example, the interruption of an intimate relationship (Slotter et al., 2010) can cause a decline in self-concept clarity. The volatility of peer relationships might undermine their influence on the maturation of self-concept clarity.

Additionally, peer relationships and parent–child relationships played unique roles in shaping the transition of adolescents’ self-concept clarity. Adolescents in the low self-concept clarity group had a higher likelihood of transitioning into the moderate or high self-concept clarity group, but neither parent–child nor peer relationships showed a significant influence on these transitions. This might reflect the profound identity confusion characteristic of adolescents in the low self-concept clarity group, who often experience heightened self-doubt and instability. Consequently, family and peer relationships might not be sufficient to effectively enhance self-concept clarity. In contrast, adolescents in the moderate self-concept clarity group were more strongly influenced by peer relationships and parent–child relationships. This may be attributed to the fact that they are in the process of gradually exploring and establishing their self-identity. Support from parent–child relationships and peer relationships can fulfill adolescents’ emotional and social needs, fostering a sense of belonging and security. This, in turn, mitigates the risk of a decline in self-concept clarity and facilitates their transition to a higher level of self-concept clarity. For adolescents in the high self-concept clarity group, enhanced parent–child relationships were found to reduce the likelihood of transitioning to the moderate group. This suggests that strong parent–child relationships provide stable support and validation, helping adolescents maintain and strengthen their self-concept clarity.

### 4.4. Implications and Limitations

We explored the developmental transition patterns and factors influencing adolescents’ self-concept clarity through a person-centered approach. The person-centered approach adopted enriches theoretical understanding by capturing individual variability in developmental trajectories, moving beyond average-level analyses and offering a more nuanced view of self-concept formation in adolescence. But the study has several limitations. First, although three waves of longitudinal data were collected, the intervals were only three months, which may be relatively short for capturing developmental changes. Future research could adopt a lengthened assessment period to examine the stability and developmental trajectories of adolescents’ self-concept clarity subgroups. Second, the study focused on the effects of peer relationships and parent–child relationships from an individual-level perspective. Future studies may consider adopting a broader ecological framework to explore the influences of multiple systems, such as school and social contexts. Third, a further limitation concerns the potential influence of gender and developmental stage. Although these factors may shape adolescents’ self-concept clarity, the present study concentrated on identifying overall profiles and longitudinal transitions rather than subgroup-specific patterns. As such, separate analyses by gender or developmental stage were not conducted. Future studies are needed to explore the subgroup differences and provide a more detailed understanding of developmental trajectories. Finally, the assessment of self-concept clarity relied primarily on adolescents’ self-reports. Incorporating multi-informant approaches or objective behavioral indicators in future research could enhance the ecological validity and strength of the results.

## 5. Conclusions

The findings reveal that adolescents’ self-concept clarity exhibits heterogeneity, with three distinct patterns consistently emerging across all three time points and heterogeneous transitions. Among these, individuals in the high self-concept clarity group demonstrate greater stability over time, whereas those in the low clarity group are more likely to transition toward moderate or high clarity profiles. Moreover, parent–child relationships exert a relatively stable influence on these developmental transitions, while the impact of peer relationships appears to fluctuate, suggesting a less consistent role in shaping changes in self-concept clarity. These results suggest several practical implications for educational and intervention practices. Attention should be paid to the inherent variability in adolescents’ self-concept clarity, recognizing that individuals may shift between profiles over time. Interventions could therefore be designed to support adolescents during periods of low clarity, helping them strengthen self-awareness and reflective capacities. Meanwhile, fostering supportive parent–child interactions and peer relationships that encourage identity exploration can strengthen the growth and maintenance of a clear self-concept.

## Figures and Tables

**Table 1:** The means, standard deviations, and correlations of the variables.

Variables	*M* ± *SD*	1	2	3	4	5	6	7	8	9
1. self-concept clarity _T1_	3.01 ± 0.64	1								
2. self-concept clarity _T2_	3.31 ± 0.67	0.49 **	1							
3. self-concept clarity _T3_	3.39 ± 0.61	0.46 **	0.63 **	1						
4. peer relationships _T1_	3.34 ± 0.75	0.08 **	0.09 **	0.10 **	1					
5. peer relationships _T2_	3.40 ± 0.71	0.08 **	0.19 **	0.18 **	0.45 **	1				
6. peer relationships _T3_	3.40 ± 0.69	0.10 **	0.15 **	0.21 **	0.40 **	0.48 **	1			
7. parent–child relationships _T1_	3.76 ± 0.81	0.31 **	0.25 **	0.24 **	0.30 **	0.20 **	0.17 **	1		
8. parent–child relationships _T2_	3.93 ± 0.77	0.22 **	0.43 **	0.36 **	0.17 **	0.35 **	0.22 **	0.50 **	1	
9. parent–child relationships _T3_	3.97 ± 0.75	0.33 **	0.33 **	0.41 **	0.17 **	0.24 **	0.37 **	0.47 **	0.57 **	1

Note: ** stands for *p* < 0.01.

**Table 2:** Latent profile analysis model fit statistics for self-concept clarity.

Time	Number of Profiles	AIC	BIC	aBIC	Entropy	LMRT	BLRT	Class Ratio (%)
T_1_	2	104,432.37	104,654.73	104,537.17	0.843	5648.96	5703.21	71.46/28.54
	**3**	**101,924.23**	**102,224.71**	**102,065.84**	**0.849**	**2510.04**	**2534.14**	**24.55/61.26/14.19**
	4	101,304.74	101,683.35	101,483.18	0.812	639.34	645.48	18.14/54.98/20.27/6.61
	5	101,004.27	101,461.02	101,219.54	0.814	323.36	326.47	2.76/20.00/18.14/52.59/6.51
	6	100,634.47	101,169.3	100,886.55	0.801	340.14	336.90	2.99/11.93/19.70/46.81/12.49/6.08
T_2_	2	91,994.98	92,213.99	92,096.43	0.891	8854.74	8769.56	63.08/36.92
	**3**	**89,547.53**	**89,843.50**	**89,684.63**	**0.863**	**2449.65**	**2473.45**	**21.20/53.35/25.45**
	4	88,297.05	88,669.97	88,469.80	0.855	1264.19	1276.47	13.67/28.91/44.29/13.13
	5	87,375.85	87,825.72	87,584.24	0.868	938.09	947.20	12.62/43.13/29.05/5.13/10.07
	6	86,986.69	87,513.51	87,230.73	0.859	585.21	590.89	12.47/9.67/21.89/41.16/9.95/5.86
T_3_	2	83,666.50	83,884.60	83,767.04	0.895	8547.65	8465.17	61.98/38.02
	**3**	**81,374.98**	**81,669.71**	**81,510.8**	**0.896**	**2295.16**	**2317.52**	**10.51/29.15/60.34**
	4	79,677.99	80,049.36	79,849.19	0.889	1706.36	1722.99	7.45/47.41/14.54/30.60
	5	78,969.41	79,417.41	79,175.93	0.895	727.49	734.58	7.19/47.41/30.60/8.76/6.04
	6	78,577.04	79,101.67	78,818.88	0.896	736.73	729.62	7.94/3.80/27.73/45.96/8.65/5.92

Note: AIC represents Akaike Information Criterion, BIC represents Bayesian Information Criterion, aBIC stands for sample size adjusted Bayesian Information Criterion, Entropy represents entropy, LMR stands for Likelihood Ratio Index, Class Ratio refers to the proportion of adolescents in each group, and BLRT represents bootstrap likelihood ratio test. Bold indicates the selected model.

**Table 3:** Latent probabilities and latent transition probabilities of self-concept clarity at T_1_, T_2_, and T_3_.

Time	Low Self-Concept Clarity Group	Moderate Self-Concept Clarity Group	High Self-Concept Clarity Group
Latent profile probabilities			
T_1_	24.55%	61.26%	14.19%
T_2_	21.20%	53.35%	25.45%
T_3_	10.51%	60.34%	29.15%
T_1_→T_2_			
low self-concept clarity group	44.40%	42.40%	13.20%
moderate self-concept clarity group	7.60%	66.50%	25.90%
high self-concept clarity group	0.40%	20.20%	79.40%
T_2_→T_3_			
low self-concept clarity group	37.60%	59.40%	3.00%
moderate self-concept clarity group	4.90%	78.30%	16.80%
high self-concept clarity group	1.70%	24.60%	73.70%

**Table 4:** Odds ratios of transition probabilities under the influence of covariates.

Time	Covariates	Low Self-Concept Clarity Group			Moderate Self-Concept Clarity Group			High Self-Concept Clarity Group		
		B (*SE*)	*OR*	95%CI	B (*SE*)	*OR*	95%CI	B (*SE*)	*OR*	95%CI
T_1_→T_2_										
low self-concept clarity group	Peer relationships _T1_	—	—	—	0.07 (0.09)	1.07	[0.90–1.28]	0.16 (0.13)	1.17	[0.90–1.51]
	Parent–child relationships _T1_	—	—	—	0.04 (0.09)	1.04	[0.88–1.23]	0.18 (0.13)	1.19	[0.93–1.53]
moderate self-concept clarity group	Peer relationships_T1_	0.02 (0.15)	0.92	[0.76–1.36]	—	—	—	0.09 (0.09)	1.09	[0.92–1.30]
	Parent–child relationships _T1_	−0.45 (0.14)	0.64	[0.49–0.83]	—	—	—	0.48 (0.08)	1.61	[1.37–1.90]
high self-concept clarity group	Peer relationships _T1_	—	—	—	0.08 (0.19)	1.09	[0.75–1.58]	—	—	—
	Parent–child relationships _T1_	—	—	—	−0.64 (0.20)	0.53	[0.36–0.78]	—	—	—
T_2_→T_3_										
low self-concept clarity group	Peer relationships _T2_	—	—	—	0.09 (0.14)	1.09	[0.84–1.42]	0.55 (0.37)	1.73	[0.83–3.59]
	Parent–child relationships _T2_	—	—	—	−0.13 (0.13)	0.88	[0.68–1.12]	0.47 (0.37)	1.60	[0.78–3.31]
moderate self-concept clarity group	Peer relationships _T2_	0.08 (0.23)	1.08	[0.69–1.70]	—	—	—	0.25 (0.12)	1.28	[1.01–1.64]
	Parent–child relationships _T2_	−0.38 (0.19)	0.69	[0.47–1.00]	—	—	—	0.50 (0.12)	1.64	[1.30–2.07]
high self-concept clarity group	Peer relationships_T2_	−0.13 (0.40)	1.14	[0.52–2.47]	−0.21 (0.12)	0.81	[0.64–1.04]	—	—	—
	Parent–child relationships _T2_	−0.66 (0.37)	0.52	[0.25–1.07]	−0.29 (0.13)	0.75	[0.58–0.96]	—	—	—

Note: Rows represent latent states at the prior time point, columns represent latent states at the subsequent time point; the reference subgroup for the dependent variable is adolescents who remained in their original group.

## Data Availability

The datasets processed and analyzed during the current study are available from the corresponding/first author upon reasonable request. The data are not publicly available due to their containing information that might compromise the participants’ privacy.
